# Supplementary material for: Microgravity Modulates Effects of Chemotherapeutic Drugs on Cancer Cell Migration
Source: Life (Basel). 2020 Aug 24;10(9):162. doi: 10.3390/life10090162 (PMC7555236; doi:10.3390/life10090162)
Supplement: Supplementary file 1 [file life-10-00162-s001.pdf]

life

MDPI

Supplementary Materials of Microgravity Modulates Effects of Chemotherapeutic Drugs on Cancer Cell Migration

Cell viability and morphometry post-microgravity and post-microgravity chemotherapy

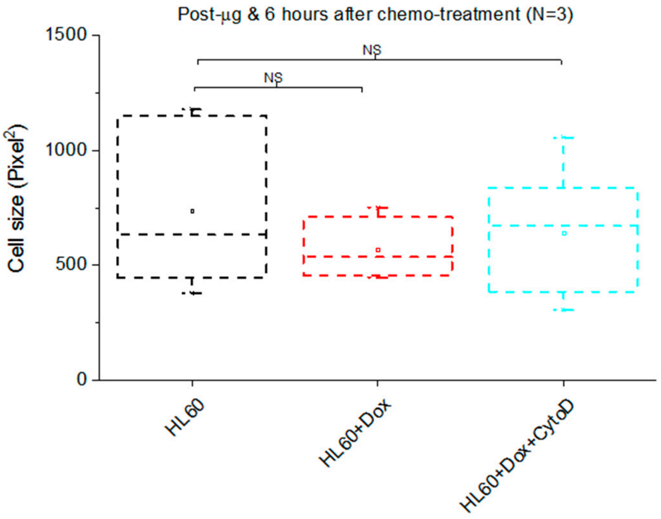

Figure S1. Box chart showing morphometric changes at 6 hours post-microgravity chemotherapy for trial 3 (N3). Although cells become smaller in size after 6 hours of incubation with Dox (5 µM) and CytoD (2 µM) as in N2 (Figure 2B) and N1, here, the reduction in size is not statistically significant (NS).

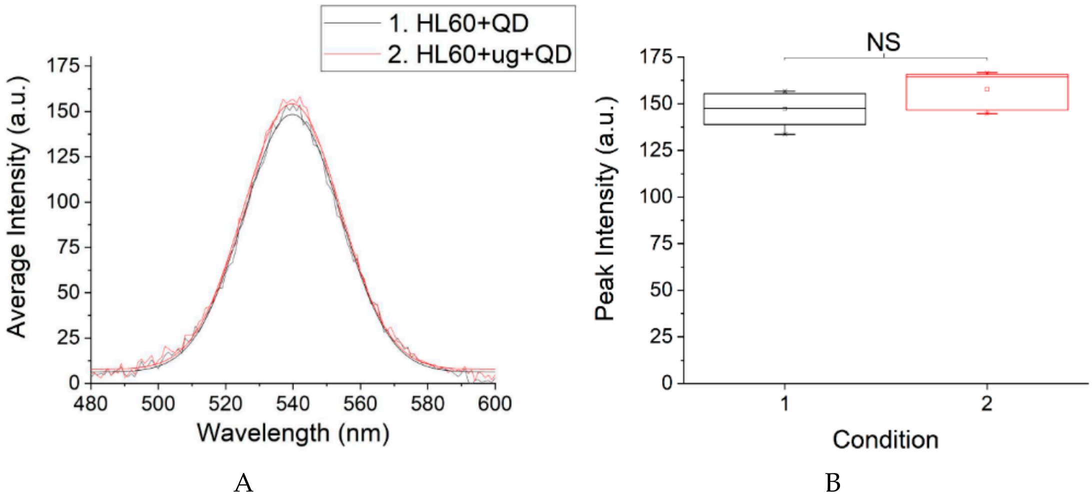

Figure S2. Assessment of ROS post-microgravity. (**A**) Quantum-dots fluorescence intensity peaks for HL60 cell suspension (HL60 + QD) and post-microgravity HL60 cells (HL60 + µg + QD). (**B**) Box chart comparing peak fluorescence intensities for the conditions in (A), showing non-significant (NS) difference based on T-Test.

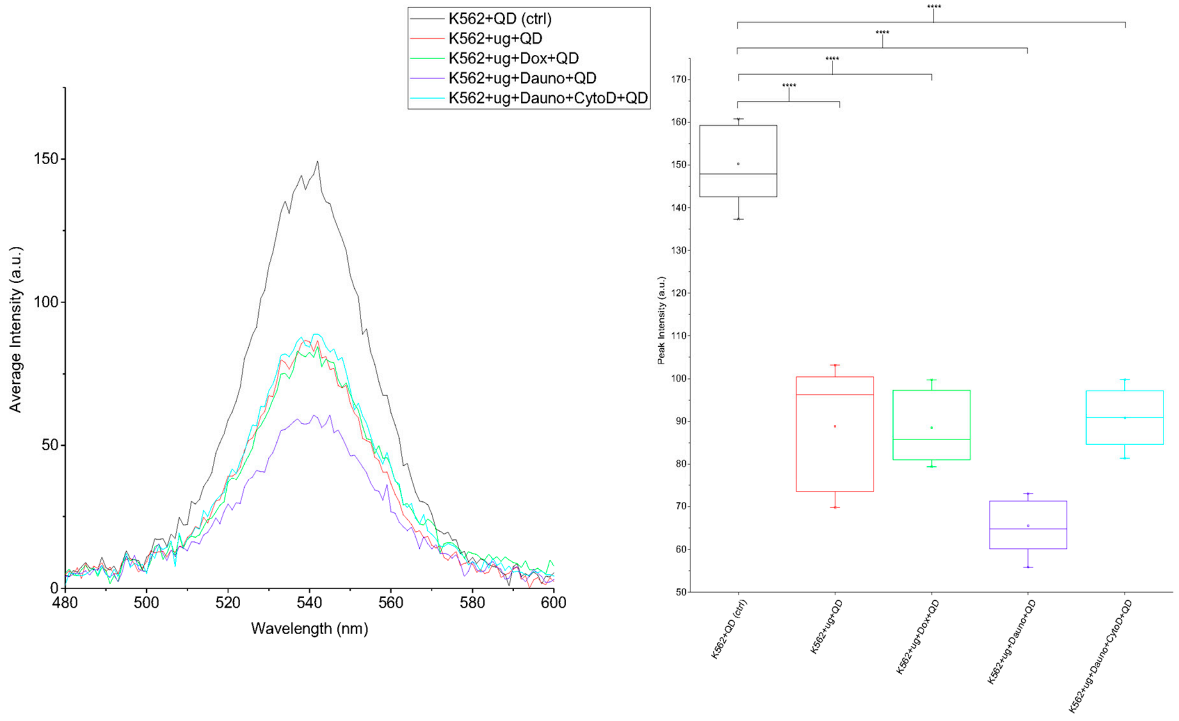

Figure S3. Quantum dots fluorescence spectra and statistical comparison for K562 cells. There are statistically significant differences (p < 0.0001 for ****) between K562 cells in 1g and K562 cells following 48 hours of microgravity (K562 + µg + QD), post-microgravity doxorubicin treatment (K562 + µg + Dox + QD), daunorubicin treatment (K562 + µg + Dauno + QD) and CytochalasinD treatment (K562 + µg + Dox + CytoD + QD).

Both doxorubicin and daunorubicin enhance post-microgravity migration of cells.

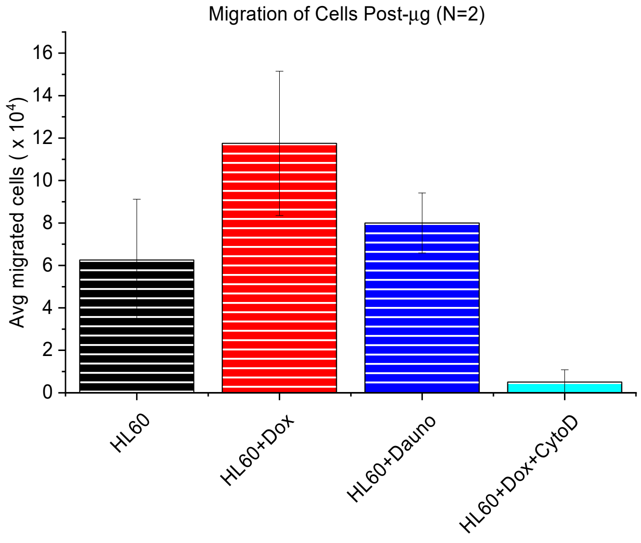

Figure S4. Post-microgravity anticancer drug treatment alters rate of cancer cell migration due to F-actin reorganization, N2. Both 5 µM doxorubicin (Dox) and 1 µM daunorubicin (Dauno) enhance post-microgravity migration of cells. The reduced migration in CytoD treated cells reveals the dependence of the migration on F-actin organization.

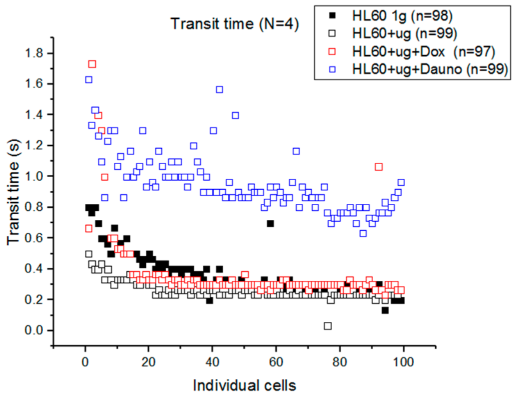

A

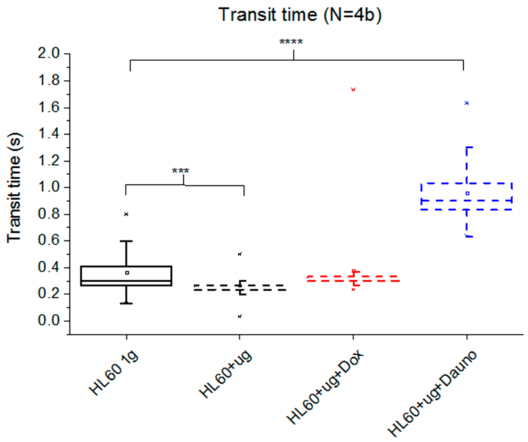

B

Figure S5. Transit times of cells advected through MMM. (**A**) Scatter plots of transit times of individual cells comparing transit times of HL60 cells, post-microgravity HL60 cells (HL60 + µg) and post-microgravity HL60 cells treated with doxorubicin (HL60 + µg + Dox) or daunorubicin (HL60 + µg + Dauno), 2 to 4 hours after treatment at a flow rate of (99 µL/hr). (**B**) Box chart comparing transit times of cells described in (A). These results fluctuated and were not consistent between various trials (N1, N2, N3, N4 and N5). However, the various controls (HL60 in 1 *g*) were consistent.

© 2020 by the authors. Submitted for possible open access publication under the terms and conditions of the Creative Commons Attribution (CC BY) license (http://creativecommons.org/licenses/by/4.0/).
